# Supplementary material for: Regulation of tert-Butyl Hydroperoxide Resistance by Chromosomal OhrR in A. baumannii ATCC 19606
Source: Microorganisms. 2021 Mar 18;9(3):629. doi: 10.3390/microorganisms9030629 (PMC8002998; doi:10.3390/microorganisms9030629)
Supplement: Supplementary file 1 [file microorganisms-09-00629-s001.pdf]

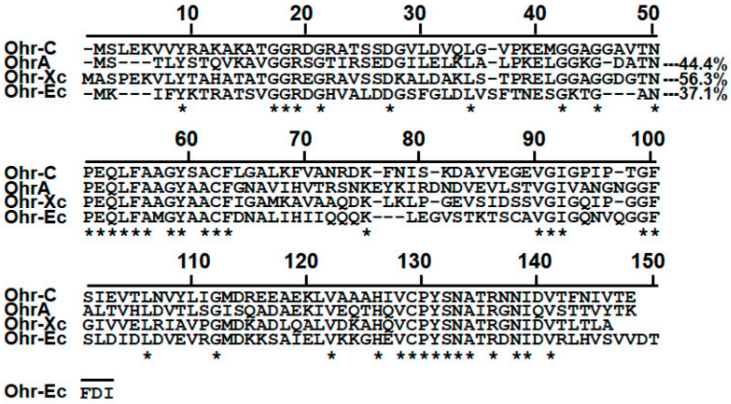

(A)

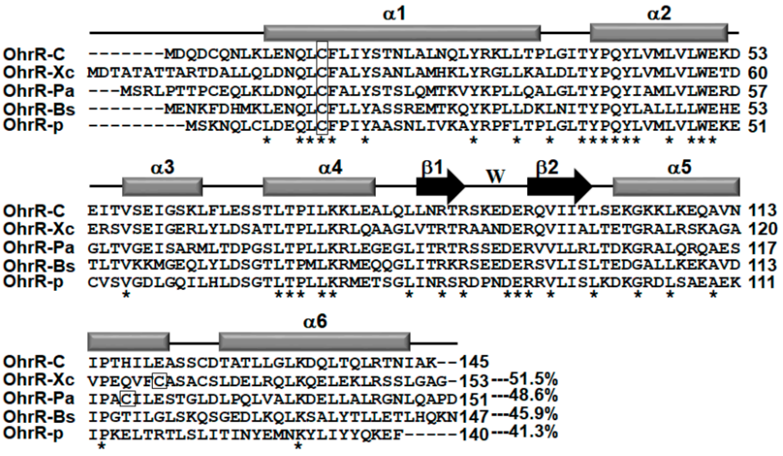

(B)

Figure S1. Amino acid identity of Ohr and OhrR proteins. (**A**) Amino acid identity of Ohr proteins. The percentages at the end of the amino acid sequences in the first row represent the level of similarity with chromosomal Ohr. Ohr-C, chromosomal Ohr; OhrA, Ohr expressed by ORF8 on pMAC; Ohr-Xc, Ohr from *X. campestris*; Ohr-Ec, Ohr from *E. coli*. (**B**) Amino acid identity of OhrR proteins. The percentages at the end of the amino acid sequences in the third row represent the level of similarity with chromosomal OhrR. OhrR-C, chromosomal OhrR; OhrR-Xc, OhrR from *X. campestris*; OhrR-Pa, OhrR from *P. aeruginosa*; OhrR-Bs, OhrR from *B. subtilis*; OhrR-p, OhrR expressed by ORF9 on pMAC.
